# Differential effects of estrogen receptor ligands on regulation of dihydrotestosterone-induced cell proliferation in endothelial and prostate cancer cells

**DOI:** 10.3892/ijo.2012.1689

**Published:** 2012-11-06

**Authors:** CHUNYAN WENG, JINGJING CAI, JUAN WEN, HONG YUAN, KAN YANG, JULIANNE IMPERATO-McGINLEY, YUAN-SHAN ZHU

**Affiliations:** 1 Department of Medicine/Endocrinology, Weill Cornell Medical College, New York, NY 10065, USA;; 2 The Center of Clinical Pharmacology; 3 Department of Cardiology, The Third Xiangya Hospital; 4 Institute of Clinical Pharmacology, Central South University, Changsha, P.R. China

**Keywords:** prostate cancer cells, androgen, estrogen, endothelial cells, cyclin A, prostate specific antigen

## Abstract

Androgen deprivation therapy of prostate cancer with estrogens shows significant cardiovascular side-effects. To develop effective prostate cancer therapeutic agent(s) with minimal cardiovascular side-effects, we compared the effects of various estrogen receptor (ER) ligands on the modulation of dihydrotestosterone (DHT) actions in LAPC-4 and LNCaP prostate cancer cells and human aortic endothelial cells (HAECs). DHT stimulated the proliferation of HAEC, LAPC-4 and LNCaP cells and induced PSA mRNA expression in LAPC-4 cells. These DHT actions were differentially modulated by ER ligands in a cell-dependent manner. In LAPC-4 cells, knockdown of ERβ expression partially eliminated the βE2 inhibition of DHT-induced LAPC-4 cell proliferation, and a parallel change was observed between ER ligand modulation of DHT-induced cell proliferation and cyclin A expression. The obtained data suggest that it is feasible to develop effective agent(s) for prostate cancer therapy with minimal cardiovascular side-effects and 17α-estradiol and genistein are such potential agents.

## Introduction

Prostate cancer is a significant health problem, accounting for approximately 900,000 new cases and more than 258,000 cancer-related deaths worldwide in 2011 (1). Currently, prostate-specific antigen (PSA) test is clinically used for early detection of prostate cancer and for surveillance of disease progression, even though it may not decrease the mortality of the disease (2). Most prostate cancers are slowly growing, but aggressive prostate cancer cases do occur, especially in cancers with a high Gleason score, and can metastasize to other sites of the body, such as the bone and lymph nodes. Thus, treatment of prostate cancer depends on the severity of the disease. For aggressive prostate cancer, the treatments are surgery, radiation therapy, hormonal therapy, chemotherapy or their combination in order to increase patients’ survival and improve their quality of life. However, there is currently no cure therapy available once prostate cancer is metastasized, and androgen deprivation therapy is one of the standard therapies (3). Since the 1940s, estrogens have been used for androgen deprivation treatment of prostate cancer as pioneered by Huggins *et al*(4). Estrogens inhibit testosterone biosynthesis through the negative feedback of the hypothalamic-pituitary-gonadal axis (5) and directly modulate androgen actions through estrogen receptors (ERs) in prostate cancer cells (6,7). However, the long-term use of estrogens in treatment of prostate cancer is limited due to their cardiovascular side-effects, such as thrombosis and cardiovascular events (8,9).

The mechanisms responsible for estrogen-induced cardiovascular side-effects are not fully understood. Several previous studies have documented that estrogens were able to directly or indirectly induce dysfunction/injury of the endothelium, resulting in thrombosis and atherosclerosis (10–12). Functionally, estrogens display their cellular and biological actions through binding to ERs (13). To date, two distinct ER isoforms (i.e., ERα and ERβ) have been identified, and several variants for each isoform have been discovered in humans or other mammals (14,15). Studies over the last decade have shown that the effects of estrogens are dependent on the receptor isoform as well as on the ratio of ER isoforms or the variants expressed in the target cells (16–18). In the cardiovascular system, estrogens can significantly impact cardiovascular functions (19,20) and ERβ may play a major role in the regulation of vascular function and blood pressure although the mechanism remains to be elucidated (21). In prostate cancer cells such as LAPC-4 and LNCaP cells, ERβ was highly expressed, while ERα was relatively low or undetectable (7,22). Thus, ERβ could mediate the direct actions of estrogens in these prostate cancer cells (6,7,23). Our previous data showed that estrogens acting on ERs produced a receptor-ligand and receptor-isoform specific modulation of androgen actions on gene expression and cell growth in prostate cancer cells (6,7,24). In this study, we further compared the receptor-ligand and receptor-isoform specificity of estrogen receptor ligands in the modulation of dihydrotestosterone (DHT) actions in prostate cancer cells and endothelial cells, aiming to develop novel therapeutic agents for prostate cancer therapy with minimized cardiovascular side-effects.

## Materials and methods

### Reagents

Dihydrotestosterone (DHT), 17β-estradiol (βE2), 17α-estradiol (αE2), diethylstilbestrol (DES), genistein and tamoxifen were purchased from Sigma Co. (St. Louis, MO, USA) and dissolved in absolute ethanol at 10^−2^ M stock solutions. 4,4′,4″-(4-Propyl-[1H]-pyrazole-1,3,5-triyl) trisphenol (PPT) and diarylpropionitrile (DPN) were obtained from Tocris Bioscences (Minneapolis, MN, USA). ICI182780 (ICI), a pure estrogen antagonist, was kindly provided by Dr A.E. Wakeling of Zeneca Pharmaceuticals (Macclesfield, UK). Reagents for real-time PCR were purchased from Invitrogen (Carlsbad, CA, USA). Antibodies against ERα (HC-20: sc-543) and ERβ (N-19: SC-6820) were obtained from Santa Cruz Biotechnology (Santa Cruz, CA, USA) and antibodies against cyclin A (cat no. C4710) and β-actin (cat no. A5316) were obtained from Sigma Co.

### Cell lines and culture

Human aortic endothelial cells (HAECs) were purchased from Lonza Walkersville Inc. (Walkersville, MD, USA) and grown in EGM-2 medium (Lonza) supplemented with 2% fetal bovine serum (FBS), hydrocortisone, human epidermal growth factor, bovine brain extract and gentamicin/amphotericin-B as described previously (25). The HAECs with less than four passages in the laboratory were used for the experiments. All experiments were carried out in the EBM™-phenol red free medium (Lonza) containing 2% stripped FBS (Gemini Bio-Products, Calabasas, CA, USA).

Prostate cancer LAPC-4 cells, an androgen-dependent cell line (a gift from Dr C. Sawyer of Memorial Sloan-Kettering Cancer Center, New York, NY, USA) were cultured in Iscove’s modified Eagle’s medium (IMEM) supplemented with 15% FBS, 2 mM L-glutamine, 1 nM R1881, 50 U/ml of penicillin, and 50 *μ*g/ml of streptomycin as described previously (7,26). R1881 was withdrawn 48 h before cell passage to conduct the experiments (6). LNCaP cells were cultured in RPMI-1640 medium (Sigma) with supplements as previously described (24). All cells were cultured at 37°C in a 5% CO_2_, 95% air-humidified atmosphere incubator.

### Cell viability assay

To determine cell viability after treatment with different steroids, HAECs, LAPC-4 and LNCaP cells were plated in 96-well plates at a density of approximately 25% in EBM-phenol red-free medium (Lonza) containing 2% stripped FBS, or in phenol red-free IMEM supplemented with 5% stripped FBS, or in phenol red-free RPMI-1640 medium supplemented with 5% stripped FBS, respectively. Twenty-four hours after plating, cells were treated with various hormones alone or in combination as indicated in each experiment. The concentrations of hormones and treatment durations were selected based on previous studies in these cells (6,24,25). The number of viable cells was determined using Cell Titer 96^®^ Aqueous One Solution Cell Proliferation Assay kit from Promega (Madison, WI, USA) according to the manufacturer’s instruction.

### Reverse transcriptase-polymerase chain reaction (RT-PCR) and quantitative RT-PCR

To determine gene expression, RT-PCR and qRT-PCR were performed. Briefly, total cellular RNA was isolated using TriPure reagents (Roche Diagnostic Inc., Indianapolis, IN, USA), and the concentration of RNA was quantified using the ultraviolet absorbance at 260 nm. cDNA was synthesized following the protocol from Invitrogen with 1 *μ*g of total cellular RNA, and PCR was carried out according to the protocol from Promega in a PCR mixture containing 1.5 mM MgCl_2_, 0.5 *μ*M of each primer, 200 *μ*M dNTPs, 2.5 units of GoTaq^®^ Flexi DNA polymerase (Promega) and 2.5 *μ*l of cDNA. The primers used are listed in Table I. The PCR conditions were 94°C for 2 min, and then 35 cycles of 94°C for 30 sec, 63°C for 30 sec for ERα or 60°C for 30 sec for ERβ, 72°C for 30 sec, and a final extension of 72°C for 5 min. The PCR products were then fractionated in a 2% agarose gel and visualized by ethidium bromide staining. pSG5-ERα and pSG5-ERβ expression plasmids were used as positive controls, and yeast tRNA was used as a negative control.

qRT-PCR was performed using the comparative Ct method according to the instructions from the manufacturer on the ABI Prism 7900 Sequence Detection System (Applied Biosystems, Foster City, CA, USA) in our institutional core facility as described previously (6). Glyceraldehyde 3-phosphate dehydrogenase (GAPDH) was used as an internal control. The difference between samples was calculated following the instructions of the manufacturer (Applied Biosystems).

### Protein extraction and western blot analysis

Protein extraction and western blot analysis were performed as described previously (25,27) with minor modifications. Briefly, LAPC-4 cells treated with various agents as indicated in each experiment were harvested for total cellular protein extraction using the passive lysis buffer from Promega. The protein concentrations were determined using the Bio-Rad Protein Assay kit following the manufacturer’s instruction (Bio-Rad, Hercules, CA, USA). Equal amounts (20 *μ*g) of total cellular proteins were fractionated on a 10% SDS-PAGE and transferred to a nitrocellulose membrane (Amersham Pharmacia Biotech, Piscataway, NJ, USA). The membrane was blocked with TBS-T buffer [500 mM NaCl, 20 mM Tris-HCl (pH 7.4) and 0.1% Tween-20] containing 5% non-fat dry milk overnight at 4°C and then incubated with specific antibodies against ERα (1:200) or ERβ (1:400) or cyclin A (1:1,000) in TBS-T buffer containing 5% non-fat dry milk for 2 h at room temperature. Following the secondary antibody incubation (1:2,000), the positive signal was visualized using the SuperSignal West Pico Chemiluminescent kit (Pierce Biotechnology Inc., Rockford, IL, USA) and exposed to Kodak X-Max film. β-actin was used as an internal control. The specific signals of ERα, ERβ, cyclin A and β-actin were quantified using Image J (NIH, Bethesda, MD, USA). The data are presented as fold changes of the control after normalizing with β-actin levels.

### Construction of small interference RNA (siRNA) and gene transfection

To knockdown ERβ expression in cells, we used ERβ siRNA and gene transfection. We first searched GenBank for human ERβ gene sequences (GenBank accession no. NM_001437) and designed a custom stealth RNAi oligonucleotide at 25 base pairs in length (Invitrogen). The sequence for ERβ was 5′-GUCAAGGCCAUGAUCCUGCUCAAUU-3′ and the control siRNA was 5′-CCAUGGCGCCAAUUCCAAACA GUUU-3′. For RNAi transfection, LAPC-4 cells were seeded in a 96-well plate or a 6-well plate in phenol red-free IMEM medium containing 5% stripped FBS without antibiotics. Twenty-four hours later, the cells were transfected with various concentrations of siRNA using Lipofectamine 2000 (0.25 *μ*l/well in 96-well plate) according to the instruction from the manufacturer (Invitrogen) in OPTI-MEM medium. Sixteen hours after transfection, transfection reagents were replaced with normal medium and cells were treated with various hormones for 72 h as indicated in each experiment. At the end of the experiments, the number of viable cells was determined using the cell viability assay described above. The efficiency of lipofectamine was tested before siRNA transfection, and the knockdown of ERβ expression was verified using western blot analysis.

### Statistical analysis

The data are presented as the mean ± SE of the mean (SEM). One-way analysis of variance (ANOVA) followed by a post hoc Student-Newman-Keuls test was used to determine the difference among multiple groups. A p<0.05 was considered as statistically significant.

## Results

### DHT induction of endothelial and prostate cancer cell proliferation and PSA expression

**Figure 1.**
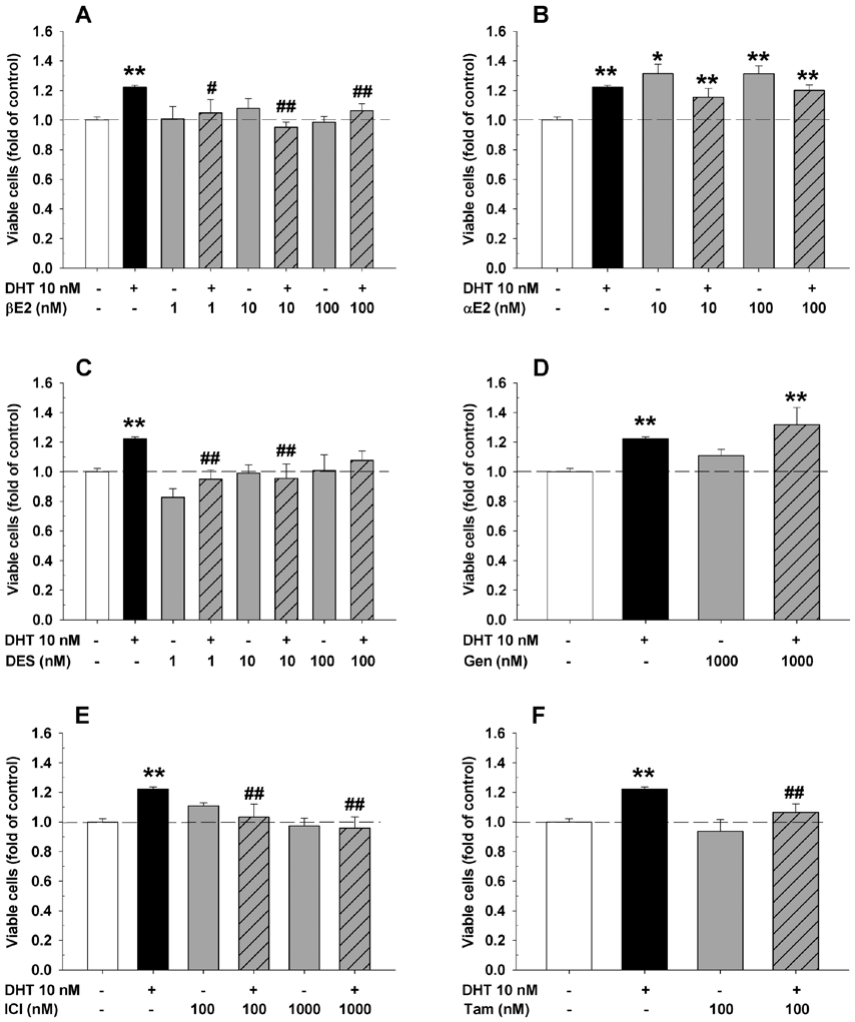
Differential effects of various ER ligands on the regulation of DHT-induced cell proliferation in HAEC cells. HAEC cells were seeded in 96-well plates and treated with or without 10 nM DHT and/or an ER ligand for 48 h. The data are expressed as fold of corresponding vehicle control of each experiment. The values are the mean ± SEM of 6–12 individual samples of 2–4 independent triplicate experiments. ^*^p<0.05 and ^**^p<0.01 compared to the corresponding vehicle control; ^#^p<0.05 and ^##^p<0.01 compared to the corresponding DHT treatment.

**Figure 2.**
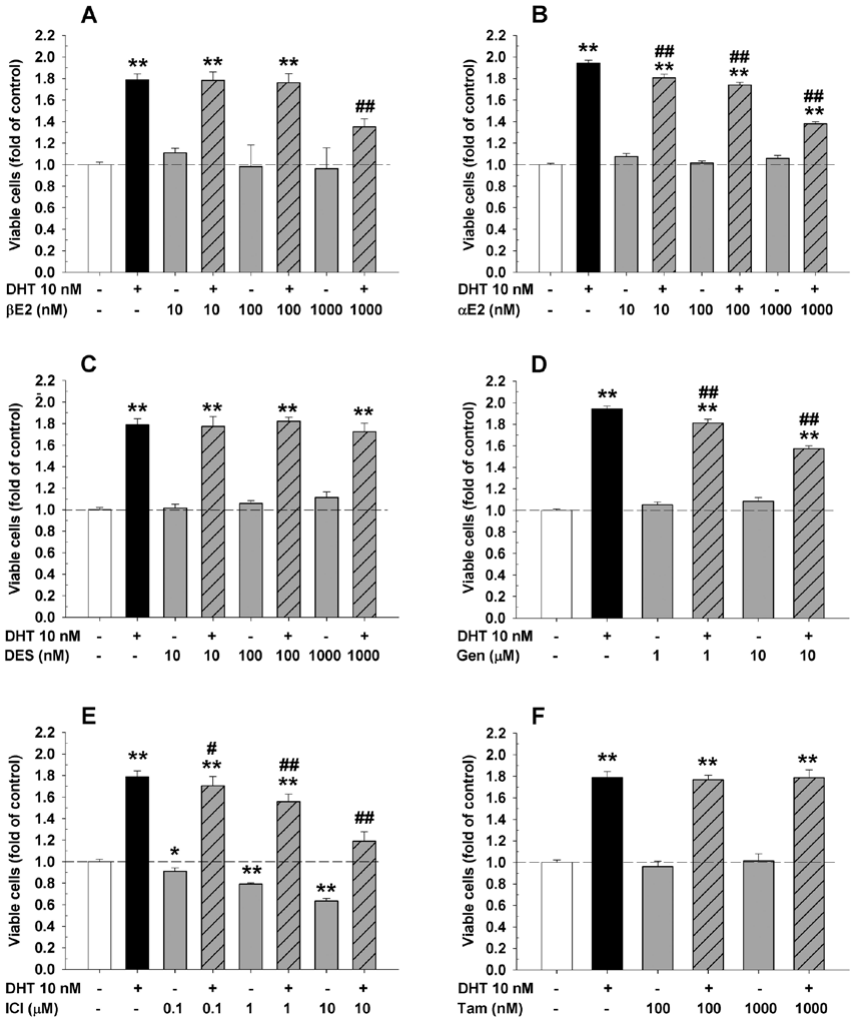
Differential effects of various ER-ligands on the regulation of DHT-induced cell proliferation in LAPC-4 prostate cancer cells. LAPC-4 cells were plated in 96-well plates and treated with or without 10 nM DHT and/or an ER-ligand for 72 h. The data are expressed as fold of corresponding vehicle control of each experiment. The values are the mean ± SEM of 9 individual samples of 3 independent triplicate experiments. ^*^p<0.05 and ^**^p<0.01 compared to the corresponding vehicle control; ^#^p<0.05 and ^##^p<0.01 compared to the corresponding DHT treatment.

**Figure 3.**
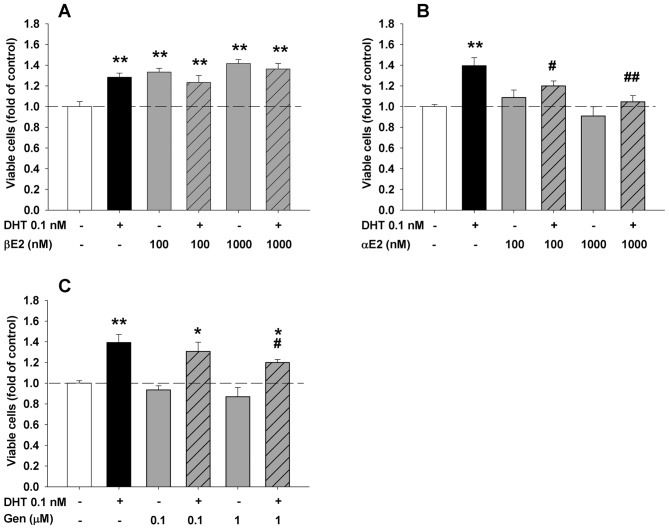
Differential effects of various ER ligands on the regulation of DHT-induced cell proliferation in LNCaP prostate cancer cells. LNCaP cells were plated in 96-well plates and treated with or without 0.1 nM DHT and/or an ER ligand for 144 h. The data are expressed as fold of corresponding vehicle control of each experiment. The values are the mean ± SEM of 6 individual samples from 2 independent triplicate experiments. ^*^p<0.05 and ^**^p<0.01 compared to the corresponding vehicle control; ^#^p<0.05 and ^##^p<0.01 compared to the corresponding DHT treatment.

**Figure 4.**
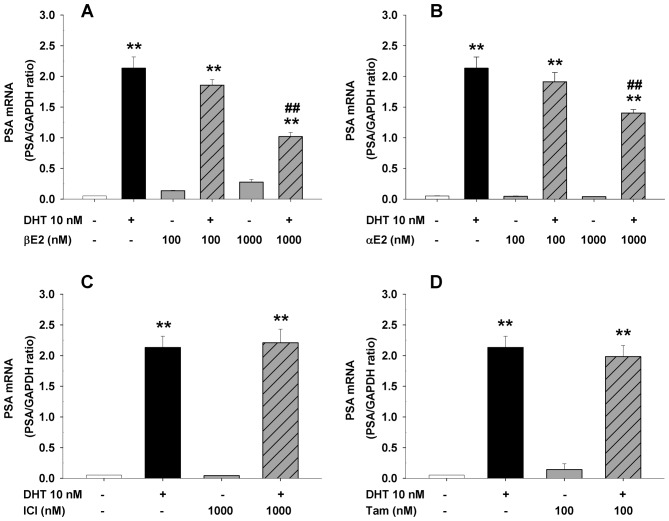
Differential effects of various ER-ligands on the regulation of DHT-induced PSA mRNA expression in LAPC-4 prostate cancer cells. LAPC-4 cells were seeded in 6-well plates at the density of 5×10^5^ cells/well and treated with the vehicle control or various doses of ER-ligands with or without 10 nM DHT for 72 h. The data are mean ± SEM of 4 individual samples from 2 independent duplicate experiments. ^**^p<0.01 compared to the corresponding vehicle control, ^##^p<0.01 compared to the corresponding DHT treatment.

In this study, we first assessed the effect of DHT on regulation of both endothelial HAEC and prostate cancer cell proliferation. We found that consistent with our previous studies (6,7,24,25), DHT significantly increased viable cell numbers of HAECs (Fig. 1), LAPC-4 (Fig. 2), and LNCaP cells (Fig. 3). Compared to the corresponding controls, cell proliferation was significantly increased to approximately 22% (48 h), 94% (72 h) and 38% (144 h) in endothelial HAECs (Fig. 1), and prostate cancer LAPC-4 cells (Fig. 2) treated with 10 nM DHT, and in prostate cancer LNCaP cells (Fig. 3) treated with 0.1 nM DHT, respectively. Moreover, treatment with DHT at 10 nM for 72 h induced PSA mRNA expression by approximately 40-fold in LAPC-4 cells (Fig. 4).

### Differential effects of ER ligands on DHT-induced cell proliferation in endothelial HAEC and prostate cancer LAPC-4 and LNCaP cells

To determine the effects of various ER ligands on the regulation of DHT-induced cell proliferation in endothelial and prostate cancer cells, endothelial HAECs or prostate cancer cells were seeded in 96-well plates and treated with DHT plus or minus various concentrations of αE2, βE2, DES, ICI, genistein, and tamoxifen (Figs. 1–3) for 48 or 72 h, respectively. As shown in Figs. 1 and 2, treatment with βE2, DES, genistein or tamoxifen alone did not significantly affect the cell proliferation of both endothelial HAECs (Fig. 1) and prostate cancer LAPC-4 cells (Fig. 2). However, administration of αE2 significantly increased cell proliferation in HAEC cells (Fig. 1B), but did not have any effect in LAPC-4 cells (Fig. 2B). Treatment with ICI alone did not significantly affect HAEC cell proliferation (Fig. 1E), but decreased LAPC-4 cell proliferation in a dose-dependent manner (Fig. 2E). In LNCaP prostate cancer cells, treatment with either αE2 or genistein alone did not affect the cell proliferation (Fig. 3B and C), while βE2 significantly increased the viable cell number (Fig. 3A).

When ER ligands were administrated concomitantly with DHT, ER ligands produced a differential regulation of DHT-induced cell proliferation in a cell type-dependent manner. For example, βE2 and ICI produced a dose-dependent inhibition of DHT-induced cell proliferation in both HAECs (Fig. 1A and E) and LAPC-4 cells (Fig. 2A and E), while αE2 and genistein significantly attenuated DHT-induced cell proliferation in LAPC-4 cells (Fig. 2B and D) without significantly altering the DHT-induced cell proliferation in HAEC cells (Fig. 1B and D). DES and tamoxifen attenuated DHT-induced cell proliferation in HAEC cells (Fig. 1C and F), but had no effect on DHT-induced cell proliferation in LAPC-4 cells (Fig. 2C and F). Similar inhibition of DHT-induced cell proliferation by αE2 and genistein was observed in LNCaP cell (Fig. 3B and C). However, the addition of βE2 did not affect DHT-induced cell proliferation since treatment with βE2 alone greatly induced cell growth in LNCaP cells (Fig. 3A).

Co-administration of ER ligands also produced a ligand-specific modulation of DHT-induced PSA expression in LAPC-4 cells (Fig. 4), consistent with our previous studies (7,24). Specifically, the DHT-induced PSA mRNA expression in LAPC-4 cells was significantly inhibited by αE2 and βE2 (Fig. 4A and B) but not by ICI or tamoxifen at the doses tested (Fig. 4C and D).

### The role of ERβ in βE2 modulation of DHT-induced cell proliferation in LAPC-4 cells

**Figure 5.**
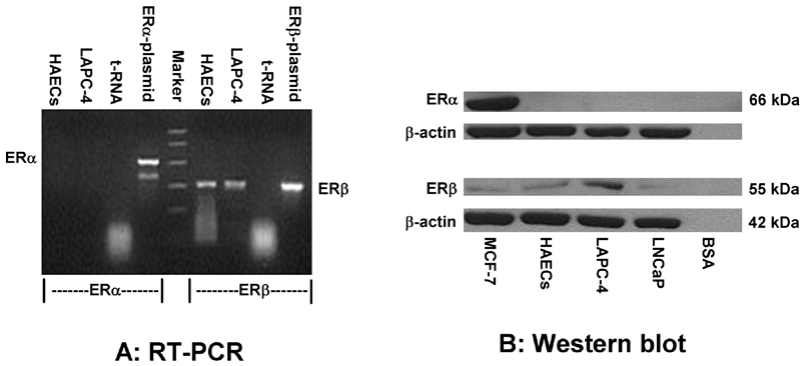
ERβ and ERα expression in HAEC and LAPC-4 cells. (A) A representative RT-PCR analysis of ERβ and ERα mRNA expression. DNA from pSG5-ERα and pSG5-ERβ plasmids served as positive controls and yeast tRNA served as a negative control for the RT-PCR. (B) A representative western blot analysis of ERβ and ERα protein expression. β-actin served as an internal control and bovine serum albumin (BSA) served as a negative control.

To investigate whether the effects of estrogens are mediated through ERs, we first determined the mRNA and protein levels of ERα and ERβ in HAEC and LAPC-4 cells using RT-PCR (Fig. 5A) and western blot analysis (Fig. 5B), respectively. As shown in Fig. 5, ERβ was highly expressed, whereas ERα expression was quite low or undetectable in both HAEC and LAPC-4 cells. As a positive control, ERα was expressed in MCF-7 cells (28) (Fig. 5B).

**Figure 6.**
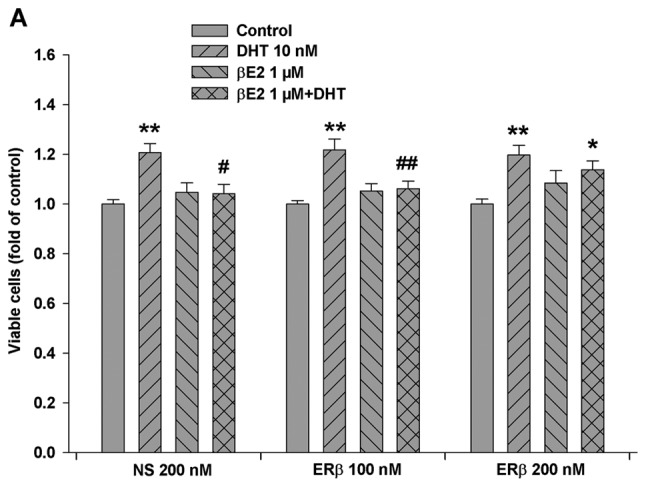

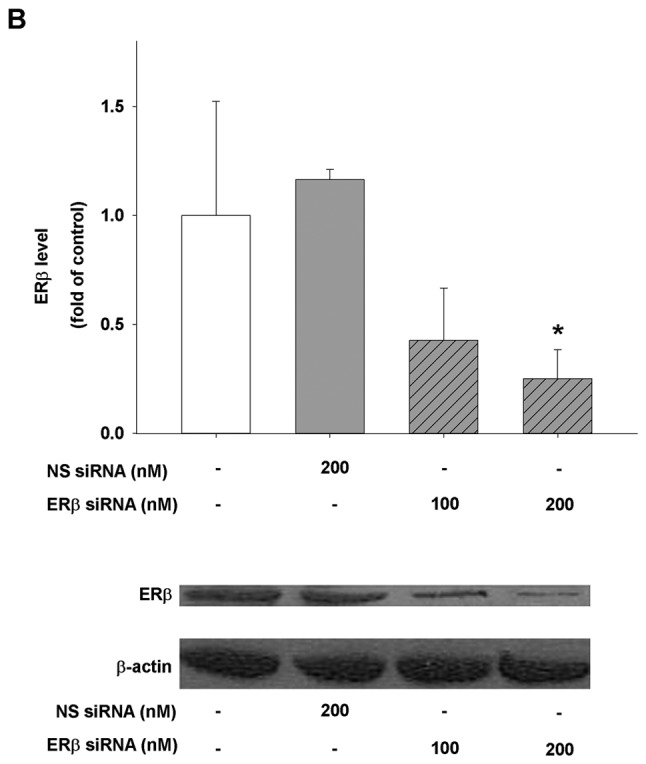
Elimination of the βE2 inhibitory action on DHT-induced LAPC-4 cell proliferation by ERβ knockdown. (A) LAPC-4 cells seeded in 96-well plates were transfected with either a specific ERβ siRNA (ERβ), or a non-specific siRNA (NS) for 16 h. The cells were then treated with the vehicle control, or 10 nM DHT, or 10 nM DHT plus 1 *μ*M βE2 for 72 h. The data are expressed as fold of the vehicle control and the values are the mean ± SEM of 4 independent triplicate experiments. (B) Knockdown of ERβ protein expression by a specific siRNA. LAPC-4 cells seeded in 6-well plates were transfected with either ERβ siRNA (ERβ) or a non-specific siRNA (NS) for 16 h, and the cells were harvested at 88 h after transfection. The quantitative data are the mean ± SEM of 4 independent western blot analyses. A representative western blot analysis is presented below the bar graph. ^*^p<0.05 and ^**^p<0.01 compared to the corresponding control; ^#^p<0.05 and ^##^p<0.01 compared to the corresponding DHT treatment.

Based on this information, we knocked down ERβ expression in LAPC-4 cells by transfection of a specific ERβ siRNA. As shown in Fig. 6, transfection of a specific ERβ siRNA produced a dose-dependent decrease in ERβ protein expression, and the knockdown of ERβ partially eliminated the βE2 inhibition of DHT-induced cell proliferation in LAPC-4 cells (Fig. 6A).

**Figure 7.**
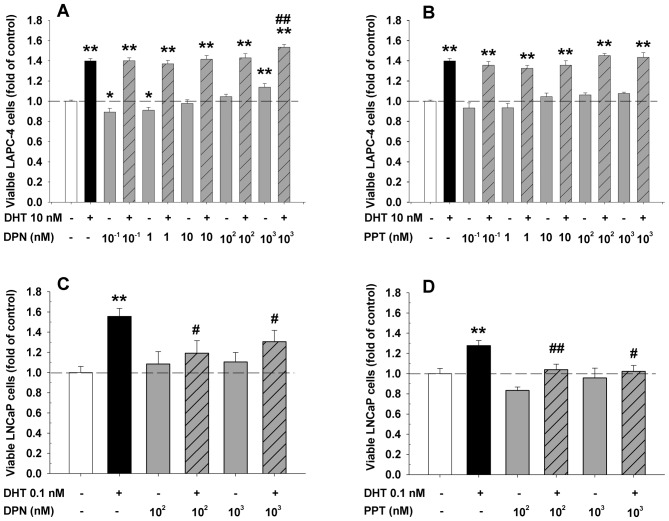
Differential effects of DPN and PPT on the modulation of DHT-induced cell proliferation in LAPC-4 (A and B) and LNCaP (C and D) prostate cancer cells. LAPC-4 and LNCaP cells were plated in 96-well plates and treated with or without DHT and various concentrations of PPT (an ERα specific agonist) or DPN (an ERβ specific agonist) for 72 (LAPC-4 cells) and 144 h (LNCaP cells), respectively. The data are expressed as fold of corresponding vehicle control of each experiment. The values are the mean ± SEM of 6–15 individual samples from 2–5 independent triplicate experiments. ^*^p<0.05 and ^**^p<0.01 compared to the corresponding vehicle control; ^#^p<0.05 and ^##^p<0.01 compared to the corresponding DHT treatment.

To explore whether a specific activation of ERβ is sufficient to inhibit DHT-induced LAPC-4 cell proliferation, the cells were treated with DHT plus or minus a specific ERα or ERβ agonist. As expected, the addition of an ERα specific agonist, PPT, failed to affect the DHT-induced cell proliferation at the doses ranging from 0.1 to 1,000 nM (Fig. 7B). Surprisingly, the concomitant administration of an ERβ specific agonist, DPN, did not inhibit the DHT action while it slightly but significantly potentiated DHT-induced cell proliferation at a 1 *μ*M dose (Fig. 7A). Of note, treatment with DPN alone produced a dose-dependent biphasic effect in LAPC-4 cells. At low doses from 0.1 to 1 nM, it slightly but significantly decreased cell proliferation while at a high dose of 1 *μ*M, it significantly increased cell proliferation (Fig. 7A). In contrast, both PPT and DPN at doses of 100 nM and 1 *μ*M completely blocked DHT-induced cell proliferation in LNCaP cells as shown in Fig. 7C and D. Treatment with PPT or DPN alone did not significantly alter the cell proliferation in LNCaP cells.

### Parallel changes in estrogen modulation of DHT-induced cyclin A expression and cell proliferation in LAPC-4 cells

**Figure 8.**
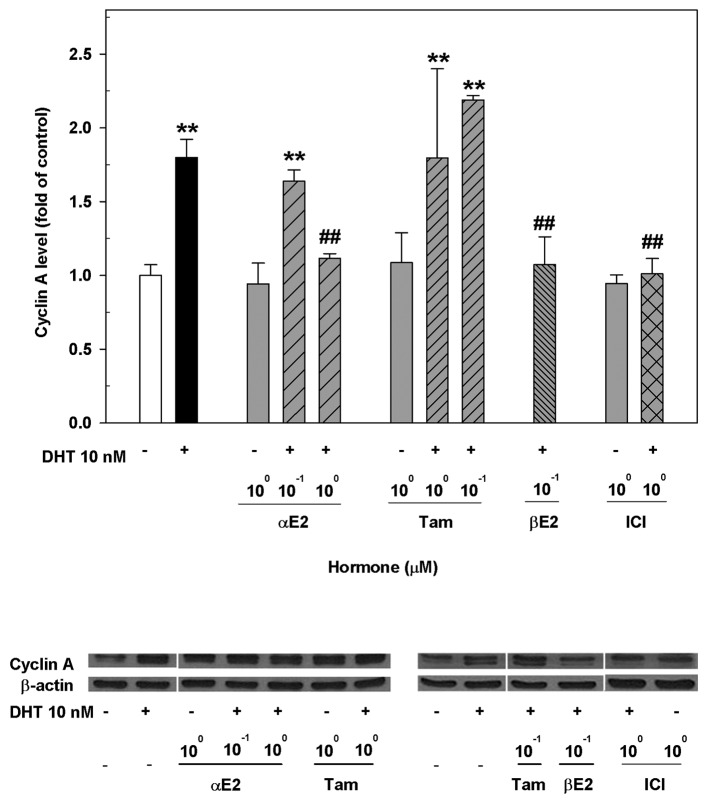
Differential effects of various ER-ligands on the modulation of DHT-induced cyclin A expression in LAPC-4 cells. LAPC-4 cells were treated with the vehicle controls, 10 nM DHT or various doses of ER-ligands alone or in combination for 72 h. The levels of cyclin A protein were determined by western blot analysis and β-actin was used as an internal control. The levels of cyclin A were expressed as fold of vehicle control and the data are the mean ± SEM (n=4). A representative western blot analysis is presented below the bar graph. ^**^p<0.01 compared to the vehicle control and ^##^p<0.01 compared to the DHT treatment. αE2, 17α-estradiol; βE2, 17β-estradiol; Tam, tamoxifen; ICI, ICI182780.

Previous studies demonstrate that cyclin A expression is induced by DHT, which is related to DHT-induced cell proliferation in both HAECs (25) and LAPC-4 cells (6). To decipher the possible molecular events responsible for the differential effects of ER ligands on the modulation of DHT-induced cell proliferation in LAPC-4 cells, we assessed the cyclin A expression after treating LAPC-4 cells with DHT and various ER ligands alone or in combination. As shown in Fig. 8, treatment of LAPC-4 cells with 10 nM DHT for 72 h significantly upregulated the expression of cyclin A. This DHT effect was significantly attenuated by the co-administration of αE2, βE2 or ICI, but not by tamoxifen at the doses used, resulting in changes parallel to the modulation of DHT-induced LAPC-4 cell proliferation (Fig. 2).

## Discussion

It has been documented that a major side-effect of androgen deprivation therapy of prostate cancer especially using estrogens is the development of thrombosis and cardiovascular events (8,9,29). The development of new therapeutic strategies and/or agents with minimal side-effects for the androgen deprivation therapy of advanced prostate cancer has been a continuing effort of the scientists around the world for the last 6 decades. With the discovery of ERβ and the elucidation of various ER ligand conformations, it is getting clearer that the effects of ER ligands are dependent not only on the receptor ligands but also on the receptor isoforms (7,16–18). Based on the recent findings that ER ligands can directly modulate androgen actions in prostate cancer cells in a receptor-ligand and receptor-isoform specific manner (6,7,24) and that androgens can directly stimulate endothelial cell proliferation in a gender-specific manner (25,30), we have compared the effects of various ER ligands on the modulation of androgen actions between endothelial HAECs and prostate cancer cells in the present study. Our data demonstrated that different ER ligands had differential effects on the regulation of DHT-inducted cell proliferation in both HAECs and LAPC-4 cells, presumably mediated through ERβ and associated with their modulation of DHT-induced cyclin A expression. These findings provide the first evidence that the effects of ER ligands in endothelial HAECs and prostate cancer cells could be dissociated and support the feasibility for the development of a novel therapeutic agent for anti-androgen therapy of prostate cancer with minimal cardiovascular side-effects.

Interactions between androgens and estrogens via their corresponding receptors play an important role in prostate and endothelial physiology and pathophysiology (6,7,31,32). The biological outcome of this hormonal interaction is not only receptor-ligand and receptor-isoform specific (6,7,25) but also cell specific as demonstrated in our current study. Consistent with previous reports (6,24,25), we have observed that treatment with DHT stimulated cell proliferation in HAECs (Fig. 1) and induced PSA gene expression (Fig. 4) and cell proliferation in LAPC-4 (Fig. 2) and LNCaP cells (Fig. 3). This DHT induced cell proliferation in endothelial HAECs and prostate LAPC-4, LNCaP cancer cells is differentially modulated by ER ligands in a cell-dependent manner. In HAECs, βE2, DES, ICI and tamoxifen blocked the DHT-induced cell proliferation, whereas αE2 and genistein did not have such effect. Interestingly, treatment with αE2 alone significantly increased the cell proliferation in HAECs, a potential beneficial effect in the endothelium to repair endothelial damage/injury. On the other hand, both αE2 and genistein inhibited DHT-induced cell proliferation in LAPC-4 and LNCaP prostate cancer cells while βE2 only attenuated the DHT-induced cell proliferation in LAPC-4 cells without any inhibition of DHT-induced cell proliferation in LNCaP cells. Moreover, treatment with βE2 alone in LNCaP cells significantly increased cell proliferation, probably via transactivation of the mutant AR in LNCaP cells (24). Taken together, our current results and previous reports strongly indicate that the modulation of DHT actions by ER ligands is receptor-ligand, receptor-isoform and cell-specific. Based on the cell specificity of ER ligands on the modulation of DHT actions, ER ligands are categorized to three different categories (Table II), which would be informative for the development of ER ligands in the treatment of prostate cancer.

It is noteworthy that the ER ligand specificity in modulation of DHT actions is unparallel or unrelated to the pharmacological categorization. For instance, ICI, a pure ER antagonist, not only blocked the DHT-induced cell proliferation in HAECs and LAPC-4 cells, but also inhibited cell growth by itself in LAPC-4 cells. Although the molecular mechanisms of ICI actions remain to be further elucidated, downregulation of AR gene expression (33) and direct inhibition of AR transactivational activity (7) may account, at least in part, for these actions. Tamoxifen, a partial ER agonist/antagonist or a selective ER modulator, completely blocked the DHT-induced cell proliferation in HAECs, but did not affect the DHT-induced cell proliferation and PSA expression in prostate cancer cells, which could partially explain the ineffectiveness of tamoxifen in the treatment of prostate cancer in clinical trials (16,34). Surprisingly, DES, an ER agonist and an agent used effectively for androgen deprivation therapy of prostate cancer in the clinic, did not display any inhibitory effect on DHT-induced LAPC-4 prostate cancer cell proliferation, whereas it completely blocked DHT-induced cell growth in HAECs at low nanomolar concentrations. These data suggest that the antitumor effects of DES may be mainly mediated through the negative feedback of hypothalamus-pituitary-gonadal axis to inhibit testosterone biosynthesis without a direct inhibition of DHT action in the tumor cells, and those patients treated with DES may be more susceptible to cardiovascular side-effects (8,9) due to its inhibition of DHT-induced endothelial cell growth.

The genomic effects of estrogens are mainly mediated through the transactivation of ERs, ERα and ERβ in the cells. Although the modulation of DHT effects by estrogens can be mediated through either ERα or ERβ as previously reported (7,35), the estrogen modulation of DHT induction of LAPC-4 cell proliferation was most likely mediated through ERβ as supported by previous studies (7,22) and our current demonstrations. In the present study, we have observed that both LAPC-4 and HAEC cells expressed high levels of ERβ mRNA and protein, while the expression of ERα was quite low or undetectable. Moreover, knockdown of ERβ expression using a specific siRNA largely abolished the effect of βE2 on the inhibition of DHT-induced LAPC-4 cell proliferation. However, an activation of ERβ by a specific ligand is not sufficient to produce inhibition of DHT actions in LAPC-4 cells since DPN, a specific ERβ agonist (36), did not inhibit, but slightly potentiate DHT-induced cell proliferation in LAPC-4 cells, further indicating the receptor-ligand specificity in the modulation of DHT actions in this system.

The observation that both PPT, an ERα specific agonist (38), and DPN, an ERβ specific agonist, significantly blocked DHT-induced cell proliferation in LNCaP, but not in LAPC-4 cells is unexpected. Like LAPC-4 cells, LNCaP cells also mainly express ERβ while ERα expression is quiet low or undetectable (7,22). Unlike LAPC-4 cells that express a wild-type AR, the AR in LNCaP cells is mutated, resulting in a wide-spectrum of ligand binding to the receptor (38). It is therefore most likely that both PPT and DPN may bind to the mutant AR and function as an AR antagonist to block DHT actions. This hypothesis is currently under investigation in the laboratory.

How different ER ligands produce a differential regulation of DHT actions in a cell-dependent manner is currently unknown. Previous studies have clearly demonstrated that different ER ligands led to different conformational changes in ERs (39–41), resulting in a differential recruitment of transcriptional factors and/or co-regulators to control the biological activity of the cells (10,37,42,43). This principle also applies in androgen-estrogen interaction (7,35). In this context, our current results suggest that based on the cell-dependent differential modulation of androgen actions by ER ligands and the elucidation of their molecular mechanisms, it would be possible to develop therapeutic agents that have great effects on prostate cancer with minimal cardiovascular side-effects. Thus, further investigation of androgen-estrogen interaction in other endothelial and prostate cancer cells, in animal models and eventually in clinical trials is warranted.

It is well documented that regulation of the cell cycle plays an essential role in cell proliferation, differentiation, and cell death (44,45). Cyclin A is a key regulator in cell cycle progression, especially in the G_1_/S transition (45). Indeed, previous studies have shown that cyclin A is overexpressed in prostate cancer cells (46) and tumor tissues (47). In the present study, we observed that DHT induced cyclin A expression in LAPC-4 cells, consistent with our previous demonstrations in LAPC-4 (6) and HAEC cells (25). Notably, this DHT-induced cyclin A expression is also differentially modulated by ER ligands in a manner parallel to their modulation of DHT-induced cell proliferation, suggesting that cyclin A might be a downstream molecular target of androgen-estrogen interaction in the control of cell proliferation.

It is worthwhile to emphasize that αE2, a stereoisomer of βE2, binds weakly to ER to form an αE2-ER complex that only transiently binds to the estrogen-responsive element (48), resulting in significantly less feminizing effects than βE2. Compared to βE2, αE2 has no carcinogenic effect in a mammalian model system (49), and has little effect on the vascular smooth muscle (50). However, αE2 can protect neuronal cells from ischemic damage as potently as βE2 (51). Unlike other ER ligands, we found that αE2 was able to specifically induce growth of HAECs, while it blocked DHT-induced prostate tumor cell proliferation and inhibited tumor growth in prostate cancer xenograft mice (5,7,24). Although the mechanism responsible for αE2 stimulation of HAEC growth remains to be determined, this αE2 action could help maintain endothelial homeostasis. Taken together, these data suggest that αE2 is superior to other ER ligands for prostate cancer therapy since it blocks AR-dependent prostate gene expression, prostate tumor cell proliferation and tumor growth, while it stimulates HAEC growth, a potential beneficial action on protection of endothelium and on minimizing cardiovascular side-effects of anti-androgen therapy.

In summary, using endothelial HAECs and prostate cancer LAPC-4 and LNCaP cells as the model system, we have demonstrated that DHT-induced cell proliferation and gene expression are differentially modulated by ER ligands in a cell-specific manner. Further exploration of this hormonal interaction in other model systems and the elucidation of the molecular mechanisms will facilitate the development of effective therapeutic agent(s) for the prostate cancer therapy with minimal cardiovascular side-effects.

## Figures and Tables

**Table I.** Primers for RT-PCR and qRT-PCR.

Gene	Primers
ERα	F: 5′-ATGAGAGCTGCCAACCTTTG-3′
	R: 5′-AGAAATGTGTACACTCCAGAAT-3′
ERβ	F: 5′-GATGAGGGGAAATGCGTAGA-3′
	R: 5′-CTTGTTACTCGCATGCCTGA-3′
PSA	F: 5′-TTGTCTTCCTCACCCTGTCC-3′
	R: 5′-CAGGGTTGGGAATGCTTCT-3′
GAPDH	F: 5′-GAAGGTGAAGGTCGGAGTC-3′
	R: 5′-GAAGATGGTGATGGGATTTC-3′

F, forward; R, reverse.

**Table II.** Classification of ER-ligands based on modulation of AR activity in HAECs and LAPC-4 cells.

Categories	ER ligands	Modulation of AR activity	
		HAECs	LAPC-4 cells
I	βE2, ICI	↓	↓
II	αE2, genistein	↑/-	↓
III	DES, tamoxifen	↓	-

↓, inhibitory effects; ↑, potentiation; -, no effect.

## Abbreviations:

DHT: dihydrotestosterone
DPN: diarylpropionitrile
ER: estrogen receptor
HAECs: human aortic endothelial cells
IMEM: Iscove’s modified Eagle’s medium
PPT: 4,4′,4″-(4-Propyl-[1H]-pyrazole-1,3,5-triyl) trisphenol
PSA: prostate-specific antigen
